# Illegal logging as a disincentive to the establishment of a sustainable forest sector in the Amazon

**DOI:** 10.1371/journal.pone.0207855

**Published:** 2018-12-05

**Authors:** Letícia Santos de Lima, Frank Merry, Britaldo Soares-Filho, Hermann Oliveira Rodrigues, Christiane dos Santos Damaceno, Marcos A. Bauch

**Affiliations:** 1 Departamento de Engenharia Hidráulica e Recursos Hídricos, Universidade Federal de Minas Gerais, Belo Horizonte, Minas Gerais, Brazil; 2 Integrative Research Institute on Transformations of Human-Environment Systems, Humboldt-Universität zu Berlin, Berlin, Germany; 3 Conservation Strategy Fund, Washington, D.C., United States of America; 4 Centro de Sensoriamento Remoto, Universidade Federal de Minas Gerais, Belo Horizonte, Minas Gerais, Brazil; 5 Serviço Florestal Brasileiro, Ministério de Meio Ambiente, Governo do Brasil, Brasília, Distrito Federal, Brazil; Austrian Federal Research Centre for Forests BFW, AUSTRIA

## Abstract

Brazil recently began granting timber concessions in public forests to promote sustainable forest use. The effectiveness of this strategy hinges on the design and implementation of the concessions themselves as well as their competitive position within the logging sector as a whole. There is, however, a lack of information on the competitive interaction between legal and illegal logging and its effects on concessions profits. We address this knowledge gap by using a spatially explicit simulation model of the Amazon timber industry to examine the potential impact of illegal logging on timber concessions allocation and profits in a 30-year harvest cycle. In a scenario in which illegal logging takes place outside concessions, including private and public “undesignated” lands, concession harvested area would decrease by 59% due to competition with illegal logging. Moreover, 29 out of 39 National Forests (≈74%) would experience a decrease in harvested area. This “leakage” effect could reduce concession net rents by up to USD 1.3 Billion after 30 years. Federal and State “undesignated” lands, if not adequately protected, could have 40% of their total volume illegally harvested in 30 years. Our results reinforce the need to invest in tackling illegal logging, if the government wants the forest concessions program to be successful.

## Introduction

Brazil has been on a long and difficult journey to legalize and manage its tropical timber sector. A key component of that effort has been the allocation of timber concessions on public lands [1–4]. However, while many technical and institutional challenges of timber concessions systems are well known [5–7], and by and large addressed in the design of concessions in Brazil, the market impacts of illegal timber production on concession success or failure have been neglected. The economic effects of illegal logging on government finances due to uncollected taxes, missing revenues, and unemployment are well recognized [8–11], but there is still a lack of information on the competitive interaction between legal and illegal logging and its impacts on the long term economic sustainability of timber concessions. Here we investigate whether the presence of illegal logging outside concessions could induce a leakage effect and, consequently decrease potential concessions profits [12,13]. We also investigate the risk of being illegally exploited that public lands face if not adequately protected.

Logging in the Brazilian Amazon has been carried out for several centuries, however the timber sector only began to flourish in the 1950s along the Amazon River targeting a handful of species mainly for the plywood export trade [1,2,14–16]. With road expansion and the settlement process careening along during the 1970s-90s, logging of old-growth tropical timber became a critical step in the frontier development process [16–18]. Timber harvest in the Amazon peaked between 1985 and 1997 when annual harvest volumes exceeded 20 million m^3^, with some years topping 40 to 50 million m^3^ [19]. From 1997–2013, harvest volumes fell to around 15 million m^3^ per year and were around 13.8 million m^3^ in 2014 [20,21]. Even at this level, timber production remains a significant influence on the landscape (at 10 m^3^ per ha, 1 million ha of forest is needed per year for this volume). The gross value product in the Amazon (accounting only for round wood) was estimated to be approximately USD 857 million in 2014 [22]. Reasons for the sector decline include unfavorable macroeconomic dynamics, command and control actions against illegal logging and partial substitution of native wood products by forest plantation and alternative materials [14,22].

Although official statistics point to a decreasing trend in the timber sector as a whole, possibly indicating success against illegal logging, this seemingly intractable problem continues to beset the industry [11,23,24]. Reports for Brazil point out that in two states, Pará and Mato Grosso (the major producers of native timber), illegal logging occurred on 78% and 54% of the total harvested area, respectively, between 2011 and 2012. In comparison to 2010–2011 the area under illegal activity in those states increased by 151% and 63% [25]. In Mato Grosso state, the total area under illegal logging in 2012–2013 was 139,867 ha [26]. A 2010 Chatham House assessment revealed a range of 34 to 95% difference between the production and consumption volumes of Brazilian timber using several data sources. The 2015 report from the same institute, using FAO statistics, found that Brazilian timber consumption volume in 2012 was by 25% higher than supplies, indicating potential illegal trading feeding the market [27]. Without command and control action, or a policy mix that includes incentive-based instruments [28], illegal logging can be more appealing than legal logging for several reasons, including the unrestricted access to timber and lower transaction costs [29].

Illegal logging affects the government and society in many ways. In addition to being a doorway to deforestation and forest degradation [16,30–33], the informal timber sector is accountable for large revenue losses for society. According to the World Bank estimates in 2004, around USD 10 to 15 Billion of revenues are lost globally every year due to illegal logging [9]. A 2015 study estimated the annual value of illegal timber trade to be around USD 17 Billion globally [27]. In addition to direct losses of revenues and uncollected taxes related to the timber market [10,34], illegal logging also creates a negative social impact through unemployment and reduced workers’ safety. Unemployment due to illegal logging is estimated to equal around 1.2 million person-days per year in the Amazon Basin [11]. Those who work in this informal economy are in a vulnerable position without formal workers’ rights [10,35] and social security taxation [8], which in Brazil altogether combine to be as high as 75% of the worker’s salary.

To promote an alternative source of legal timber production and catalyze a sustainable timber sector, the Brazilian government approved a Federal Law in 2006 granting timber concessions within public forests [36]. Harvesting in Brazilian timber concessions finally began in 2010 with three forest management units in National Forests. Within these limited concessions only 160,000 m^3^ were harvested between 2010 and 2013 and the government earned a total value of USD 6.6 million. As of 2014, there were 513,000 hectares under concession [22,37]. Eight additional National Forests and one “undesignated land”–areas belonging to the government but without any determined specific use–have been identified in 2015 as suitable for establishing new concessions, totaling around 2.3 million hectares that could be converted into forest management units [22]. A decade after the law was approved, however, the concession program has not reached its full potential [29]. Key problems in the roll-out of the program have been a complicated program design and the overwhelming bureaucracy in implementation, which may constitute a disincentive for loggers [29]. Recent changes and adaptive management, however, have retained the potential for the concession program to be a significant part of a successful timber sector. While production from these areas hinges partially on the design and implementation of the concession process, effectiveness is also subject to the competitive position of concession production within the timber market, which in the case of Brazil may mean competing directly with illegal production for market share.

Here we present the results of scenario modelling simulations that quantify the potential impacts of illegal logging on concessions profit through leakage [12,13]–here referred as a displacement of logging to outside concessions induced by restrictions inside concessions. We quantify these effects on National Forests and in Federal and State Undesignated Lands through a spatially explicit economic model [2] adapted with a new set of algorithms that allow us to differentiate two harvest modes that serve as proxies for legal and illegal logging. The model estimates economic returns to logging for any location in the Brazilian Amazon, allowing for a regional analysis through a spatial resolution of 1 km^2^. We assume that illegal logging is accomplished through “conventional” logging (CL) practices, which can be thought of as the uncontrolled and unplanned cutting of selected trees [30–32,38]. CL increases timber supply, therefore competing in the market with legal logging due to the avoidance of planning, taxation and control. Legal logging is assumed to be Reduced Impact Logging (RIL), and includes a significant component of planning to maximize efficiency while minimizing impacts [39,40]. The norms and practices adopted by the Brazilian government for timber concessions can be considered to imply RIL [41], through rules of maximum harvest intensity, adoption of forest management units, annual cutting areas and protection of areas against re-entry during the harvest cycle [40]. We compare two harvest scenarios simulated for one 30-years cycle, described as:

- LEGAL scenario. RIL is adopted not only in National Forests and in other Sustainable Use Lands, but also in private and undesignated public lands, and represents what could be considered a fully functioning forest sector with legal logging on all available sites. In this scenario undesignated lands are transformed into legal working forests;
- ILLEGAL scenario. RIL occurs inside the National Forests and other Sustainable Use Lands, but now with competition with CL in private lands and undesignated public lands representing the business-as-usual trends in which all land outside protected sustainable use areas allows conventional, or illegal, harvest.

## Materials and methods

### Model setup

**Fig 1:**
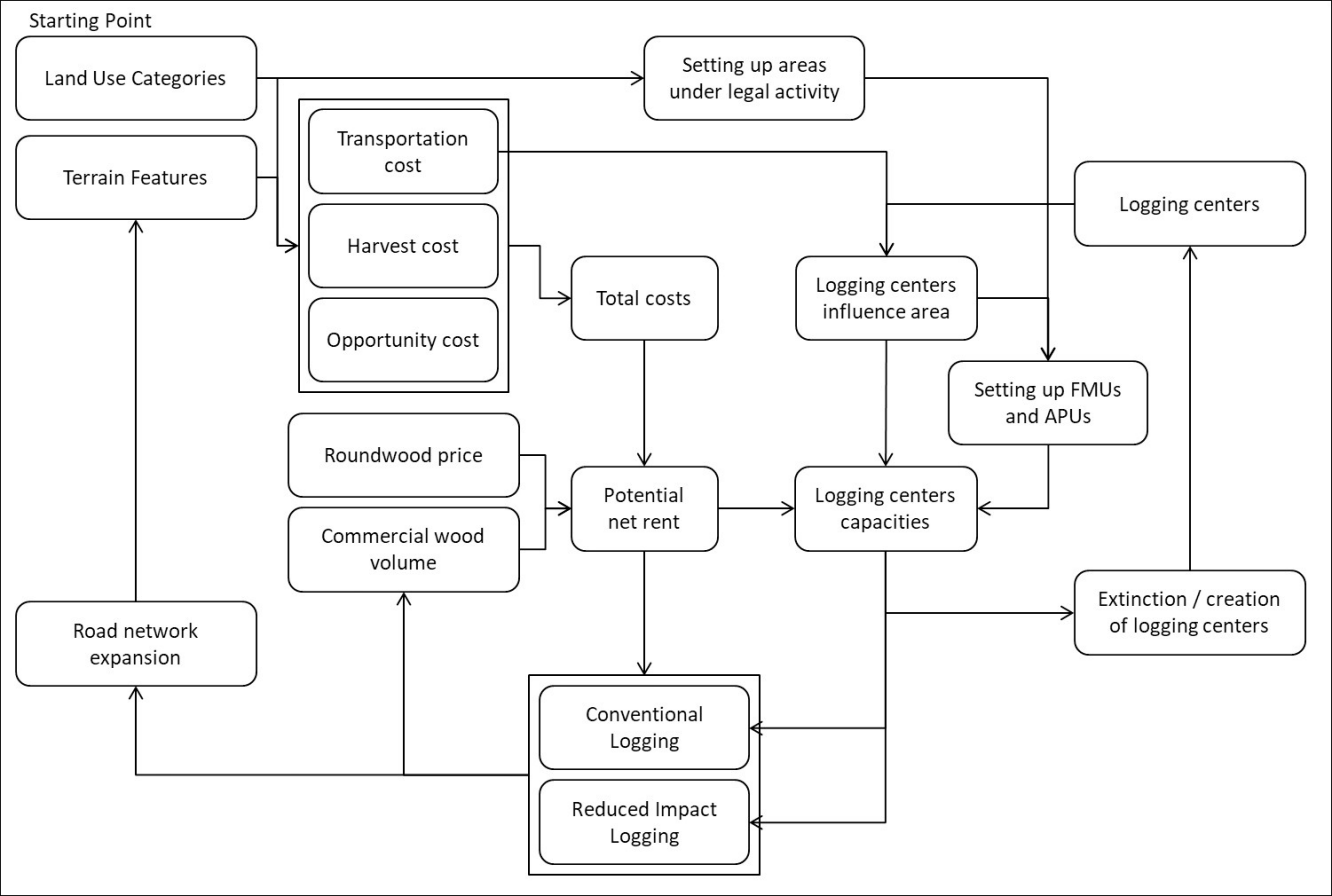
Main model components and processes. FMU stands for "forest management unit" and APU stands for "annual production unit".

Our model builds upon earlier work developed to assess the expansion of the timber industry in the Amazon that has been used in several economic analyses of land use and land use change [2,42,43]. The original model itself builds upon early simulation work [44]. The current version was developed in Dinamica EGO freeware [45]. We have improved the model platform, updated the data where possible and added a functionality to address the difference between CL and RIL (Fig 1). Our work is based only on estimates derived from previous field campaigns carried out either by researchers or NGOs, and official data provided by the government. Therefore, we disregarded any assumption not supported by ground data. The model runs annually for a 30-year harvest cycle beginning with 2009 as the baseline year. We selected 2009 because it was the latest year with the largest amount of updated information about the logging sector in the Amazon, including specific studies regarding prices of wood performed by IMAZON [18,46]. Base maps cover the total area of the 9 Federal States of the Brazilian Amazon with a spatial resolution of ~1 km^2^, comprising a total area of around 516 Million hectares, of which 302 Million were considered forest area in 2009 according to PRODES/INPE, after raster resampling to the model spatial resolution [47]. Table 1 summarizes parameter values used in the simulations.

**Table 1:** Model parameters.

Constant	Unit	Value
Spatial resolution	decimal degree	0.09
Initial year	-	2009
Number of simulated years	Year	30
Harvest cycle	Year	30
Initial capacity of newborn logging centers	m^3^year^-1^	50,000
Maximum logging intensity (RIL mode)	m^3^ha^-1^year^-1^	0.86
Forest management unit (FMU) size (RIL mode)*	ha	27,000
Annual production unit (APU) size (RIL mode)*	ha	900
Temporal variation of round wood price	% year^-1^	0
Maximum potential increase in logging center capacity	% year^-1^	20
Minimum distance to an emerging logging center	km	200
Maximum distance to an emerging logging center	km	500

* RIL = reduced impact logging.

### Categories of protected areas

According to the Brazilian Law n. 9985/2000 (National System of Nature Conservation Units), National and State forests are a subset of the Sustainable Use Lands which are public lands that may be subject to a variety of sustainable uses, including traditional use by local communities, harvest of natural resources under certain rules, and research by scientific groups. Each public forest must have a management plan that determines allowable use. Forest concessions for timber harvest are allowed in selected National and State Forests after an annual government harvest plan [22] and through bidding processes.

"Undesignated Lands" are lands that belong either to the Federal or the State Government. They are public lands without any official designation and are mostly located in remote areas of the Amazon, particularly in the state of Amazonas. Although they cannot be possessed by any private owner without a proper judicial process, they have often been exposed to indiscriminate land-grabbing. To control this situation, the Federal Government has, in some cases, undertaken an official settlement process or assigned them to other designation, such as National Forests or Strictly Protected Areas. There continues to be concern that if these lands are not soon designated, they will continue to be an easy target for illegal logging and deforestation [48].

We use the Public Forests National Registry–CNFP [49] as the source for our land zoning maps in order to differentiate the categories of protected areas. The main zoning map contains the following major categories: (a) Non-protected land, (b) Sustainable Use Lands, (c) Strictly Protected Areas, (d) Undesignated Lands, and (e) Military Areas. Additional zoning maps are used to locate and identify National and State Forests (sub-category of Sustainable Use Lands), and Indigenous Lands. These maps are used (a) to model scenarios, one in which public forests are put under strict protection, and another in which they become concessions; and (b) to identify and differentiate the areas under sustainable forest management (RIL mode) from those under conventional logging (CL mode).

### Commercial wood volume

The initial commercial wood volume map is calculated from a biomass map and vegetation parameters [2, 50, 51]. Wood volume is then estimated in cubic meters per hectare (m^3^/ha) from the biomass data (S1 Fig). In order to update the map to account for deforestation, we remove commercial wood volume from cells where deforestation had occurred up to 2009 using a reclassified land cover map by PRODES–Amazon monitoring system by satellite [47].

### Timber demand and logging capacity

Demand for timber is established using logging centers (pre-established milling capacity at the nearest processing hub). The logging centers correspond to municipality seats that had logging activity during the baseline year (S2 Fig). Following Merry et al. [2], they were defined based on the combination of the municipal seat map and national statistics for round wood yield per municipality (m^3^/year), both provided by IBGE–*Instituto Brasileiro de Geografia e Estatística*, the official authority for statistics and demography in the country [20]. We define the initial capacity of each center as the total amount produced in the baseline year. Once initial logging centers’ capacities are defined (m^3^/year), they can evolve depending on the amount of profitable wood available in its area of influence [2]. If there is more profitable commercial volume than the current capacity, the logging center can increase it up to a certain limit per year, which is set in this simulation to 20% (Table 1). If capacity equals to the total profitable volume available, it will decrease in the next time step to follow the available volume (S3 Fig). Decreasing capacity following the decrease of profitable volume can cause a center to shut down. The model is then set to search for nearby areas with high profitable volume to create up a new center, following industry rationale of migrating logging centers as observed in the Brazilian Amazon [2].

### Timber prices

We then estimate the volume of profitable timber by setting a round wood price at the sawmill and subtracting all production costs until we are left with a residual price for the standing tree (stumpage). We used a weighted average round wood price (R$/m^3^) for each logging center and its area of influence (S2 Fig). The price was weighted based on three classes of wood and their estimated representation in production in 2009 by IMAZON, which constitute the best estimates available for that year [52]. For those municipalities not contemplated in that study, we used the IBGE estimates from 2009 for round wood gross rent divided by the volume produced in the same year per municipality [20]. Most of the values matched expected average prices, but some of them were below a threshold derived from our observed values, thus they were discarded and replaced by average prices calculated by using all observations. Importantly, we assume that prices and costs of production are constant through time. These are strong assumptions but necessary in a study area bereft of data and other economic analyses that would supply elasticity estimates.

### Transportation, harvest, and capital costs

Transportation costs–expressed in R$/m^3^/km–reflect the effort of crossing each type of terrain (or land use). For example, on roads and other transportation routes, it refers to the cost of transporting a cubic meter of round wood per kilometer [2]. On land designated as Strictly Protected Areas (PA) or Indigenous Lands, the value is set to represent a crossing barrier [53] (Table 2). Total transportation cost of each cell–the least cost pathway–represents the distance from the forest to the logging center (assigned within each area of influence of a logging center) multiplied by the transportation cost across cell types. The area of influence is set according to the transportation costs to the nearest logging center [2,53] (S2 Fig). We created a transportation network–including roads and waterways–from the National Plan for Logistics and Transportation–PNLT [54]. Secondary roads network is updated every time step following new harvested areas. Roads and waterways are classified by their traffic conditions in the baseline year (paved, unpaved, planned, two-lane roads, navigable, non-navigable, inexpressive navigation waterways). In order to account for barriers, such as non-navigable rivers, apart from those presented in PNLT map, we use two other sources: the flow accumulation map from HydroSHEDS [55] and a local source map called "Hidrografia Bifiliar" [56]. These sources were then combined with the land use map derived from PRODES [47]. The zoning maps are used in order to differentiate barriers according to the category of land protection.

**Table 2:** Transportation costs.

Category	Unit	Value
**Land cover**		
Deforested	-	0.7
Grasslands	-	0.7
Forest	-	0.8
Non-navigable water bodies	-	2.0
**Public forest type**		
Sustainable use	-	0.8
Undesignated lands	-	0.8
Strictly protected areas	-	5,000
Military area	-	5,000
Indigenous lands	-	5,000
**Roads and waterways**		
Paved road	R$m^-3^km^-1^	0.3
Unpaved road	R$m^-3^km^-1^	0.5
Duplicated road	R$m^-3^km^-1^	0.2
Navigable rivers	R$m^-3^km^-1^	0.1
Seasonally navigable rivers	R$m^-3^km^-1^	1.0

R$ is the Brazilian currency.

Harvest costs include felling and skidding round wood, and then loading trucks at the harvest site. We adopt harvest costs reported by IMAZON [52] for the major logging centers in the Amazon. For the centers not contemplated by this study, we used the average value from the same source. Opportunity cost of capital is represented by an average interest rate. As in Merry et al. [2] we use the value of 5% year^-1^. We set this value in order to be conservative about the impacts of the variables on a long-term simulation model (a higher interest rate would have negated any economic value within the model time frame). Total costs are the sum of transportation, harvest and opportunity cost for each cell of the study area map and are updated every time step.

Total costs are assigned as follows.

$${\mathrm{Total}\;\mathrm{Cost}=(\mathrm{HC}}_{\mathrm{center}}{+\mathrm{TC}}_{\mathrm{ij}}{)*(1+\mathrm{Int}}_{\mathrm{rate}})$$ (1)

Where HC_center_ represents the harvest cost within the area of influence of a logging center, TC_ij_ represents the total transportation cost from cell_i,j_ to a logging center within its respective area of influence, defined by the lowest costs to all centers, and Int_rate_ is the interest rate [2,42].

We were unable to include in this version the cost of potential fines or the “risk of being caught” that illegal loggers face. Although this is highly relevant, as far as we are aware, there is no empirical study that could be used to estimate this cost. In addition, we were unable to find any research focused on fines and the costs of illegality that could be used to give us adequate ground data.

### Harvest modes

In order to compare the two types of logging practices, we adopt a set of basic rules to differentiate RIL from CL. Areas where legal logging occurs (RIL mode) are harvested according to the Brazilian rules for sustainable forest management. One of the rules is that the area must be divided into forest management units (FMUs) which are then divided into annual production units (APUs) (S4 Fig). The number of APUs inside a FMU equals to the number of years of the harvest cycle (Table 1). Here we assume that sustainable forest management has a harvest cycle of 30 years, and therefore each FMU is divided into 30 APUs. According to the Brazilian legislation, CONAMA resolution n. 406/2009 [7], the maximum logging intensity is set at 0.86 m^3^/ha/year multiplied by the number of years of the harvest cycle. Therefore, for each year of the cycle the maximum timber volume allowed for harvesting is 25.8 m^3^/ha (0.86*30 years). For each FMU, one APU area is harvested per year while the rest is left untouched. Each APU will be harvested just once per cycle, allowing the area to recover (S5 Fig). In contrast, areas under CL are not divided in production units, therefore enabling logging to occur driven only to the most profitable available areas and according to the center capacity. The total available commercial volume can then be fully harvested (if center capacity allows), disregarding maximum logging intensity. Furthermore, there is no rule constraining the return of the logger to the same area to harvest again in the case there is some commercial volume left. Strictly Protected Areas, Indigenous Lands and Military Areas are not subject to logging in these simulations (Table 3).

**Table 3:** Assumptions made for the LEGAL and ILLEGAL scenarios.

	LEGAL	ILLEGAL
Harvest in National Forests?	Yes	Yes
RIL or CL in National Forests?	RIL	RIL
Harvest in Undesignated Lands?	Yes	Yes
RIL or CL in Undesignated Lands?	RIL	CL
Harvest in Private Lands?	Yes	Yes
RIL or CL in Private Lands?	RIL	CL
Harvest in other Sustainable Use Protected Areas?	Yes	Yes
RIL or CL in other Sustainable Use Protected Areas?	RIL	RIL
Harvest in Strictly Protected Areas, Indigenous Lands, Military Lands?	No	No

### Timber harvest dynamics

Logging net rent (profitability) is calculated as the difference between the gross rent and total costs for each map cell. Gross rent is calculated multiplying the commercial wood volume available to be harvested from each map cell by the average round wood price in the region. For areas under RIL, the gross rent is affected by the maximum logging intensity regulated by law, and the availability of areas to be harvested is constrained by the annual production units. The most profitable available areas in each simulation time step (S6 Fig) are harvested according to the rules (RIL or CL) previously assigned for each area, their profitability and the capacity of each logging center. The available commercial wood volume changes over time as the harvest takes place in the simulation. No tree regrowth is considered since the model runs for only one harvest cycle. The model continues to harvest annually until available profitable timber is less than demand at which time the logging center capacity declines at a rate commensurate with the available profitable timber (S3 Fig). During the annual harvest process new roads are built and the costs of access and transport of logs further into the forest continually decline. Simulation of unpaved road construction follows the least cost pathway and the correspondent transportation cost is applied (Table 2). Selected cells to be logged are connected to the logging center by existing roads combined with the new unpaved ones that are built to reach these areas (S7 Fig). Maps of road network, wood volume, cost surface, profitability, and area of influence are then updated for each annual iteration.

### Scenarios

Table 3 presents the scenario assumptions in terms of the areas where harvesting is allowed and the harvesting mode, i.e. reduced impact logging (RIL) or conventional logging (CL). All other settings and inputs were kept constant for both scenarios.

### Model and data uncertainties

As in any type of modeling study, there are uncertainties that must be considered while interpreting and using the results. In particular, the simulations are constrained by the reduced amount of detailed and spatially explicit data available for the Amazon timber sector. In addition, technical aspects related to biomass mapping and spatial resolution of input data are of concern, as detailed below:

1. The model was conceived and developed for a regional analysis and therefore it is limited in its ability to evaluate local processes in detail. The most important constraint is the spatial resolution of the maps used as inputs (≈1 km^2^), which are not appropriate for a local analysis. For instance, total forest area in the baseline year, calculated after raster conversion from PRODES/INPE dataset to the spatial resolution used in this model corresponds to 302.45 Million ha, while in the PRODES original dataset it corresponds to 297.88 Million ha [47], which means an error of around 1.5%. Spatial resolution also constrains representation of FMUs and APUs in terms of size and formats. Although they are represented as squares in the model, actual FMUs can have more complex shapes as they might have rivers and roads as boundaries.
2. The commercial wood volume map is based on a remote sensed biomass map [50]. Biomass cannot be measured directly through remote sensing and this fact introduces some uncertainty. Up to now, consensus on large-scale biomass values is not easily reached in science [57–60]. Mitchard et al. [60] compared two different remote sensed biomass maps with interpolated maps derived from a large number of biomass measurement ground plots. They observed a difference of more than 25% between remote sensing maps and observed values in ground plots. However, doubts remain on whether using interpolated maps from ground measurements would solve the problem. Although the network of forest plots in the Amazon encompasses more than 400 points [60], the Amazon region is very large and there is a huge internal variation on forest structure, which would require many more forest plots in order to characterize it properly to produce large scale biomass maps.
3. The model estimates wood demand based solely on the current and projected capacity of the logging centers. This means that no projections on international or national demands for timber are taken into account in the model [2,42].
4. We assume that the only rule governing the choice of loggers for the areas to be harvested is the cost-benefit balance–expressed in terms of potential net rents–constrained by the land use rules established by the government. However, many other local factors, including social-cultural ones, interact to drive the loggers' behavior and their choices.
5. Prices are taken as fixed throughout the entire harvest cycle. This is due to our inability to accurately forecast timber prices as well as other input prices that would also affect rents (for instance, fossil fuel prices affecting transportation costs, and so on).
6. Wood prices are estimated as a weighted average value (proportion of volume by class of wood and average prices per class) due to the lack of standardized data for species, class, and density in different regions.

## Results

### Brazilian Amazon timber production

**Fig 2:**
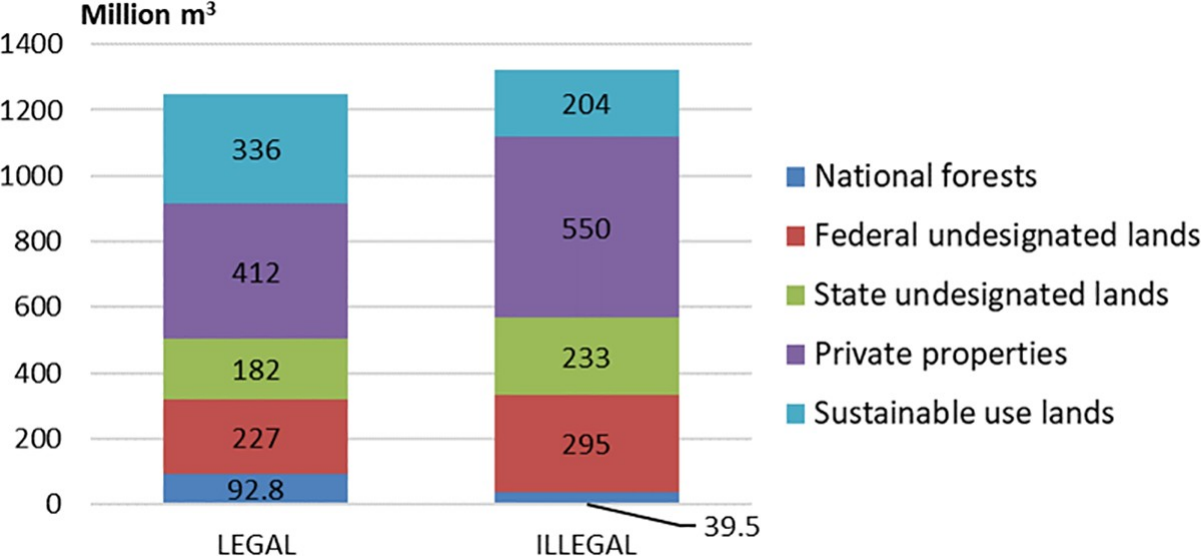
Contributions of different land categories to total simulated timber volume harvested in the Brazilian Amazon. Comparison of LEGAL and ILLEGAL scenarios for a 30-years cycle production (Million m^3^).

We estimate a commercial wood volume baseline (year 2009) equal to 4835 Million m^3^ for the Brazilian Amazon, distributed in a total area of around 302 Million ha of forests. Under the LEGAL scenario, the total simulated volume harvested during the 30-years cycle would be equal to 1250 Million m^3^ (Table 4). Logging would affect a total area of 84.6 Million ha and would generate a total net profit of USD 45.1 Billion. Under the ILLEGAL scenario, logging would produce 1321 Million m^3^ during the 30-years cycle, affecting a total area of 91.1 Million ha and generating a total net profit of USD 53.6 Billion. Therefore, in comparison to the LEGAL scenario, the ILLEGAL scenario would represent an increase of 7.7% in total logged area, 5.7% in total logged volume, and 18.8% in total profits generated by the sector. Contributions from different land categories would also change according to the scenarios. In comparison to the LEGAL scenario, the ILLEGAL scenario would result in decreased production of Sustainable Use Lands and National Forests while increasing production in private properties, State and Federal Undesignated Lands (Fig 2). The major contributors to the total timber volume in both scenarios would be private properties with 412 and 550 Million m^3^ produced in the LEGAL and ILLEGAL scenarios, respectively (Fig 2). The second major contributor would be the Sustainable Use Lands category in the LEGAL scenario (336 Million m^3^) and the Federal Undesignated Lands in the ILLEGAL scenario (295 Million m^3^).

**Table 4:** Baseline (year 2009) commercial wood volume stock (m^3^) and total area (hectares) for Brazilian Amazon States, National Forests, Federal and State Undesignated lands, and non-protected areas. Comparison of LEGAL and ILLEGAL scenarios after 30 years of simulated harvest cycle: total volume harvested (m^3^), total logged area (hectares) and total net profit (USD).

	2009—Baseline		LEGAL—30 years cycle				ILLEGAL—30 years cycle	
	Commercial stock volume	Total area	Total volume harvested	Total logged area	Total net profit	Total volume harvested	Total logged area	Total net profit
	Million m^3^	Million ha	Million m^3^	Million ha	Million USD^†^	Million m^3^	Million ha	Million USD^†^
Brazilian Amazon^‡^	4,835	516	1,250	84.6	45,116	1,321	91.1	53,594
National Forests	223	14.5	92.8	5.4	2,859	39.5	2.2	1,579
Federal Undesignated lands	428	39.7	227	15.6	9,044	295	20.2	12,478
State Undesignated lands	586	37.5	182	9.4	6,134	233	14.1	9,841
Private Properties	792	215	412	36.1	16,911	550	45.9	23,314
Sustainable Use Lands^§^	871	56.6	336	18.1	10,168	204	8.7	6,382

^†^Average Dollar Price (USD) in Brazilian Currency (BRL) in 2009: R$1.995.

^‡^Comprises the area of the 9 Brazilian Amazon States. From this total, forest area in the baseline year corresponded to 302 Million ha.

^§^Public forests designated for sustainable use from different land categories (excluding National Forests and Indigenous Lands).

### Leakage effect in National Forests

**Fig 3:**
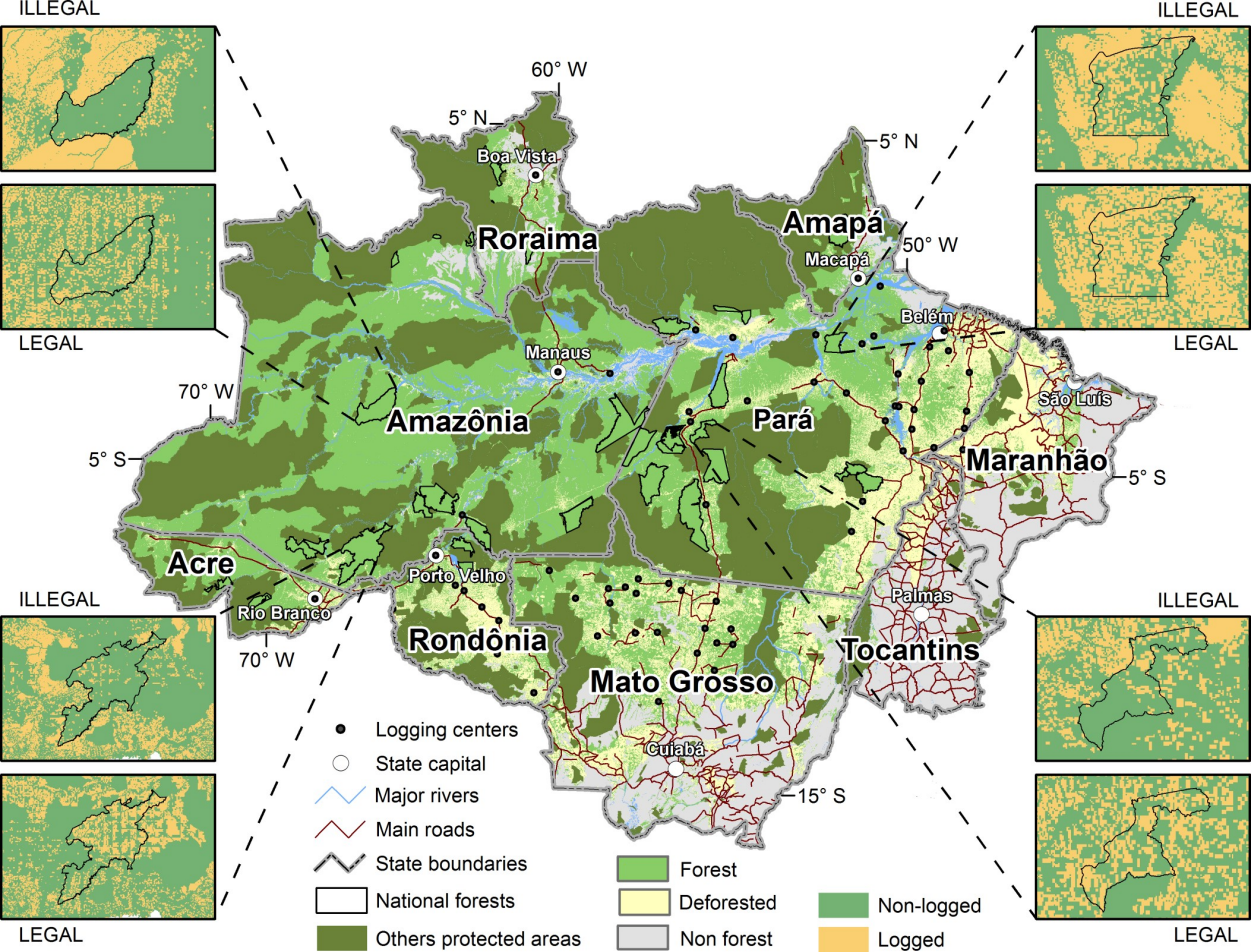
Logged area in four National Forests after 30 years of harvesting. Comparison of LEGAL and ILLEGAL scenarios. Logged areas represented in orange color. Outputs from the logging model (see Methods) implemented in Dinamica EGO software version 3.0.5 (Dinamica EGO Copyright 1998–2015 Centro de Sensoriamento Remoto / Universidade Federal de Minas Gerais–Brazil. Available at http://www.csr.ufmg.br/dinamica). Map design made in ArcMAP version 10.2 (ArcGIS Copyright 2016 Environmental Systems Research Institute, Inc. http://desktop.arcgis.com/en/arcmap/).

**Fig 4:**
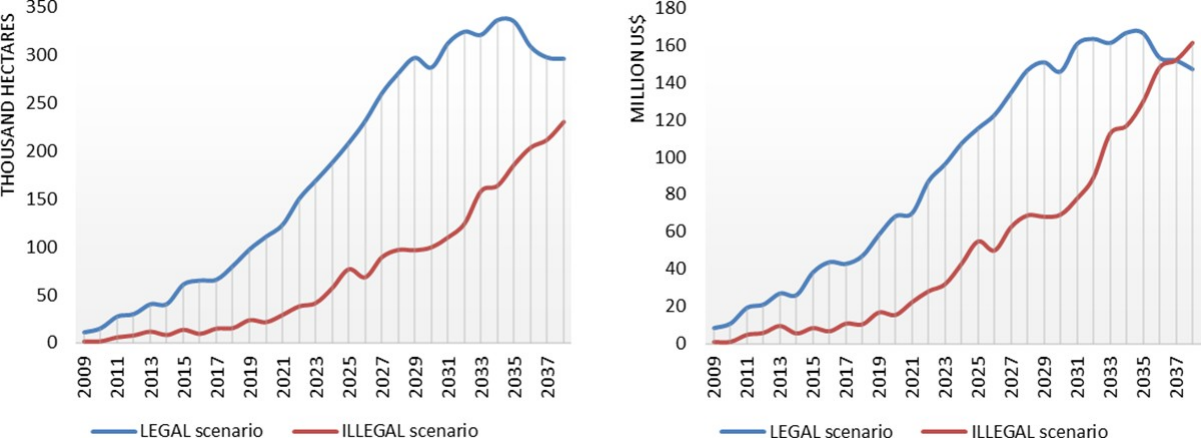
**Simulated RIL logged area [Thousand hectares] (A), and profits [Million USD] (B), only inside federal concessions under the LEGAL and ILLEGAL scenarios.** Profits are calculated according to 2009 exchange rate USD/BRL. The LEGAL scenario results are represented in green and ILLEGAL in brown.

The presence of illegal logging outside concessions reduces the total area harvested inside concessions in National Forests. Under the LEGAL scenario, total area logged inside the National Forests during the 30-years harvest cycle would be 5.4 Million ha. The corresponding volume harvested would be equal to 92.8 Million m^3^. In contrast, under the ILLEGAL scenario, total area logged in National Forests would reach only 2.2 Million ha and total volume harvested would be equal to 39.5 Million m^3^ (Table 4). The total logged area map (Fig 3) depicting the results of the two scenarios shows a leakage effect for four National Forests. Total logged area in National Forests would decrease by 59% (Fig 4A) with losses for 29 out of 39 National Forests (S8 Fig). This leakage effect could reduce total profits generated by concessions from USD 2.9 Billion (LEGAL) to around USD 1.6 Billion (ILLEGAL) (Table 4). This means losses of up to USD 1.3 Billion in 30 years for the Federal Government. Profits earned inside concessions under the ILLEGAL scenario is estimated to be higher than that of the LEGAL scenario just in the 3 last years of the harvest cycle (Fig 4B), possibly due to lack of timber resources outside concession after many years of CL. In addition, expansion of roads to reach other timber sources would progressively decrease transportation costs, favoring exploitation in National Forests just in the final years of the harvest cycle. In terms of contributions for the Brazilian Amazon timber sector as a whole, the proportion of logged area in National Forests in relation to the total logged area in the Amazon would drop from 6.3% (LEGAL) to 2.4% (ILLEGAL). While under the LEGAL scenario, total volume produced in National Forests represents 7.4% of the total produced in the Brazilian Amazon, this contribution decreases to 3% under the ILLEGAL scenario. At the same time, there is an increase in volume contributed from private properties from 33% (LEGAL) to 41.6% (ILLEGAL), suggesting a displacement to those areas.

### Spatial and temporal patterns of timber production

The simulated scenarios generate different spatial and temporal patterns of logging. Until 2020, under the ILLEGAL scenario, the states of Pará and Mato Grosso are the leading timber producers (S9 Fig). However, the State of Amazonas overtakes both states by 2023 and 2020, respectively. Amazonas follows a steep trajectory of timber production, reaching 43 Million m^3^ in 2036 in case illegal logging is not halted. Under LEGAL scenario, however, logging in the state of Amazonas would reach 33.8 Million m^3^ in 2038 and the trajectory would be smoother (S10 Fig). This difference seen for the Amazonas State (almost 10 Million m^3^) would be due to the presence of a large area of Undesignated Lands that would be targeted by illegal loggers (those lands comprise around 30% of the total area of the Amazonas State). The simulations under the ILLEGAL scenario presents a decline in Pará State timber production starting in 2019, while the LEGAL scenario shows that under sustainable forest management Pará could keep increasing production up to 2030. This difference seems to be related to the effect of restrictions of volume and harvest area imposed by law in the LEGAL scenario, while in the ILLEGAL scenario, most of the logging activity would affect every profitable wood volume available. Under the ILLEGAL scenario, Mato Grosso State follows a steep decline after 2021, while under LEGAL scenario it follows a smooth trajectory all over the 30-years harvest cycle, losing precedence for Amapá State just in 2030. The ILLEGAL scenario also points out that Acre State would be the third largest producer after 2031, surpassing Mato Grosso and Amapá, due to the decline of wood availability in the other federal states and the increasing access through new unpaved roads.

### Vulnerability of Undesignated Lands to illegal logging

The Undesignated Lands are especially vulnerable to illegal logging, but could play a major role in sustainable timber production if adequately managed [3,61]. Under the ILLEGAL scenario, and thus in the absence of command and control actions to prevent illegal logging, Undesignated Lands would be targeted by illegal loggers progressively through the simulated harvest cycle. Just in Federal Undesignated Lands, illegal logging could affect up to 20.2 Million ha during the 30-years cycle, approximately 55% of the total area of this category (Table 4). This would correspond to 295 Million m^3^ of timber illegally harvested in 30 years (69% of the baseline commercial wood volume of this land category). Total foregone revenues from illegally harvested trees would be approximately USD 12.5 Billion. In turn, State Undesignated Lands would have 233 Million m^3^ illegally harvested (40% of its baseline volume) from an area of 14.1 Million ha (38% of its total area). Under the LEGAL scenario, assuming that Undesignated Lands would be designated for sustainable use following the Brazilian regulation for logging practices, the Federal Undesignated Lands would produce 227 Million m^3^ of timber in 30 years from an area of 15.6 Million ha (39% of its total area). In turn, the State Undesignated Lands would produce a total volume of 182 Million m^3^ from an area of 9.4 Million ha (25% of its total area) (Table 4). Together, Federal and State Undesignated Lands would contribute with 33% of the total timber produced in the Amazon under the LEGAL scenario. In contrast, under the ILLEGAL scenario, they would contribute with 40% of the total volume harvested in the Amazon, but without revenues for the Government. However, under the same ILLEGAL scenario, if Undesignated Lands were converted into fully protected areas, there would be an overall decrease of 30% in the total volume harvested in the Amazon during the 30-years cycle (around 400 Million m^3^). The difference from the contribution of Undesignated Lands to the total production in the ILLEGAL scenario (40%) to the simulated decrease in overall Amazon production if they are fully protected (30%) is due to an effect of displacement of illegal activity to other areas, compensating part of the inaccessibility caused by the protection of undesignated lands.

## Discussion

The negative impacts of illegal logging can affect ecosystem services [30,38,62], local communities [11] and the Government revenue [8,34]. Between 2011–2012, in Pará State, around 67% of the total area exploited by illegal logging was private, undesignated or disputed lands [63]. Thus, isolated strategies for timber concessions in public forests alone do not suffice to promote a sustainable timber sector given that land tenure issues may play, as well, an important role in illegal logging.

Competition between illegal activity outside concessions and sustainable forest management inside concessions implies high economic losses for the Brazilian Government. The uncontrolled logging in the surroundings of National Forests reduces the chances of legal logging being economically attractive due to the difference in the access to timber outside and inside those public lands. We demonstrate this by comparing two scenarios (ILLEGAL and LEGAL) in which an estimated revenue of USD 1.3 Billion is lost from the timber concessions in National Forests. We show that if logging inside and outside public concessions follows the same legal rules (LEGAL scenario)–adopting forest management plans and avoiding overharvesting–then concessions become more attractive for formal loggers (Figs 3 and 4, S8), and the government gains much more with the price of their standing trees.

In addition to forgone revenues in the timber concessions due to leakage, uncollected taxes over timber from illegal sources, and access of illegal loggers to Undesignated Lands economically affect government and society. Although overall timber profits would increase under the ILLEGAL scenario during the 30-years cycle (Fig 2), the Government would only benefit from revenues from timber concessions in National Forests and indirectly through taxation over timber produced in other Sustainable Use Lands, losing revenues from taxes not applied in the informal sector. In contrast, under the LEGAL scenario, government would benefit from taxation over the whole production in the Amazon. Our simulation results also suggest that contributions of National Forests and other Sustainable Use Lands to the overall timber production of the Amazon would be higher, were illegal logging not present outside those areas (Fig 2).

Excessive bureaucracy and lack of opportunities for capacity building may discourage under-prepared actors to participate in the concessions system and hence may drive some actors to the informal sector. To counteract this, the public bidding process for forest concessions should be tailored to facilitate the participation of small-scale loggers in order to become attractive to local entrepreneurs [29]. As it is currently designed, it may constitute a high risk for small companies to bear the transaction costs. In addition, more in-depth studies focused on the capacity of small landowners to comply with the law regarding sustainable forest management in private lands would support an evaluation of the effectiveness of the current policies targeting non-industrial actors.

Under the concession system, command-and-control actions are still needed. According to SFB monthly bulletin [64], from November 2017 to June 2018, among three of the six national forests with timber concessions (Jamari, Caxiuanã, and Saracá-Taquera), at least one was subject to illegal logging each month. In addition to illegal timber harvest, some concessions were targeted by illegal miners. Concessionaires have been notifying authorities and command-and-control actions have been undertaken.

Despite big losses for environmental conservation, the revised Forest Code of 2012 (FC) introduces new mechanisms to address fire management, forest carbon, and payments for ecosystem services, which could bring environmental benefits. More importantly, it establishes an online land registry system (CAR) that streamlines the process for landowners to register their property environmental information [65]. As of September 2018, 96% of Brazil’s 5.5 million rural properties were in system. The CAR dataset transparency, together with advanced remote sensing techniques for mapping selective logging [66], is of particular importance for licensing and monitoring logging in private properties, given that the FC allows and regulates sustainable forestry management in Legal Reserves, i.e. the portion of native vegetation that all properties must set aside as a conservation reserve (from 80% in the Amazon to 20% in the other biomes). Therefore, enforcement of the FC through CAR documentation and monitoring plays a key role in fostering a sustainable forest industry not only outside concessions but also inside them given the interlocked effect of illegal logging outside concessions on their economic sustainability.

Undesignated Lands are particularly vulnerable to illegal logging and deforestation [48]. With ineffective control of illegal logging, natural capital in the form of available wood contained in large patches of rainforest located in the Undesignated Lands could be drastically reduced over the years by overharvesting. According to our simulations, up to 69 and 40% of the baseline timber volume from Federal and State Undesignated Lands, respectively, could be subject to illegal logging in 30 years. This represents an impressive reduction of timber stocks primarily belonging to the government, and a threat to biodiversity. It is just a matter of time for having spontaneous roads expanding into those remote areas; hence entailing profits for illegal loggers inside Undesignated Lands, and consequently, government losses of timber stocks [61]. As roads are progressively built throughout the landscape access costs are reduced, exposing Undesignated Lands to illegal logging, especially in the State of Amazonas due to the high density of commercial wood. Those forests not only represent timber resources but also areas that are rich in non-timber forest products [67], high biodiversity [68–72], large carbon stocks [50,58,73–74], and are strategically linked to regional precipitation patterns [75,76]. Protecting all these natural resources would require assigning those Undesignated Lands an adequate designation and taking additional actions to curb illegal logging [48]. Particularly, fully protecting these lands would be highly relevant for Brazil’s commitments with the Paris Agreement, given that they currently represent vulnerable areas for illegal deforestation [77–78].

Although scenario modelling is a suitable way to study the effects of illegal logging on the Amazon timber sector, achieving a complete LEGAL scenario in practice is a far-fetched goal due to the inherent complexity of social, economic and political conditions surrounding the use of natural resources in the Amazon. Monitoring, command and control actions are expensive and may not suffice to halt illegal logging. Complementary strategies must be therefore considered, not only related to logging *per se*, but also to land grabbing issues and related illegal deforestation [78]. A policy mix that puts together command and control actions and incentive-based programs should therefore be pursued in order to achieve a comprehensive conservation strategy for the Amazon [28]. In addition, it is important to look into the supply-chain dynamics and its pitfalls [79]. A variety of instruments has been tested in different countries aimed at controlling land use changes, such as product certification, moratoria, and commodity roundtables [80]. These instruments may constitute a potential integrated strategy to curb illegal logging in the Amazon. However, to succeed, careful attention should be also placed on strategies that counteract illegal tactics, such as frauds that have been detected in the government’s timber licensing system as reported by Greenpeace [81]. These frauds are a central component of illegal trading aimed at international consumers, such as US and Europe [82–83].

Our study highlights the potential interactions between illegal and legal logging that may produce a disincentive for sustainable harvest inside concessions and ultimately, in every other place where legalized timber harvesting could take place. As far as we are aware, it is also the first one to estimate the impact of the competition between illegal and legal logging using large-scale scenario simulation. Nevertheless, our model results include uncertainties inherent to any modelling exercise, as discussed in the Materials and Methods Section. Particularly, we are aware that the model is framed around a cost-benefit analysis, and as such, leaves out complex social processes that may impact the timber sector. Further improvement in the model will be achieved with data from the national forestry inventory carried out by the Brazilian Forest Service aimed at mapping timber species. These data could be complemented with new biomass estimates, timber prices, harvest and transportation costs, international and national projections of wood demand, legal costs and fines, and the implementation of additional algorithms to assess the effect of individual and collective behavior of loggers on the sector as a whole.

## Conclusion

Logging concessions in public forests have the potential to generate revenues for the Government that can be used to fund protected areas and increase social benefits such as environmentally sound local employment. This seems to be the logic followed by the Federal government nowadays, through the Brazilian Forest Service. A number of requirements made by the government included in the bidding processes aim at the generation of local employment, access of local communities to collect non-timber forest products and protection against other types of land exploitation, e.g. mining. Therefore, the public concessions, *if correctly managed*, have the potential to support better working conditions in the logging sector, increase social security taxation, Government revenues, and as a result, to improve the local economy. However, illegal logging outside concessions may represent great disadvantage for legal loggers through competition and can induce losses of Government revenues as shown in our study. In order to ensure effectiveness in the long-term, the Federal Government must keep concessions profitable and attractive for legal loggers, and one of the key actions would be tackling the illegality outside them. Both forest protection and profits for the Government, therefore, are linked to a comprehensive conservation planning. This includes legal designation of public lands, expansion of the protected area network, land tenure certification, and economic incentives for legal timber trade. Harnessing such policies is therefore mandatory to ensure the success of the Amazon logging concessions.

## Supporting information

S1 Fig Volume of available commercial wood in the Amazon (m3/ha) and logging centers. Calculated based on Merry et al. (2) (co-authors) updated with official land cover map of 2009 from PRODES/INPE (3) (Available at http://www.dpi.inpe.br/prodesdigital/prodes.php?LANGUAGE=EN&). Map design made in ArcMAP version 10.2 (ArcGIS Copyright 2016 Environmental Systems Research Institute, Inc. http://desktop.arcgis.com/en/arcmap/).
(TIF)

S2 Fig Example of logging centers and their areas of influence in Rondônia State, Brazil. Logging centers are depicted in black spots and areas of influence with a variety of shades of green. This is an output from the logging model (see Methods) implemented in Dinamica EGO software version 3.0.5 (Dinamica EGO Copyright 1998–2015 Centro de Sensoriamento Remoto / Universidade Federal de Minas Gerais–Brazil. Available at http://www.csr.ufmg.br/dinamica). Map design made in ArcMAP version 10.2 (ArcGIS Copyright 2016 Environmental Systems Research Institute, Inc. http://desktop.arcgis.com/en/arcmap/).
(TIF)

S3 Fig Total logging center capacity following the profitable available wood volume in the model. Center capacity is expressed in Thousand m^3^ per year (red line), following the profitable available wood volume in Thousand m^3^ per year (blue line). Graph created using Microsoft Excel 2010.
(TIF)

S4 Fig Example of division of FMUs into 25 APUs. FMUs are represented as large squares with orange borders while each APU is represented by a small square.
(TIF)

S5 Fig Selection of one APU per FMU. FMUs are represented as large squares with orange borders while each APU is represented by a small square. Grey small squares represent the selected APU for each FMU.
(TIF)

S6 Fig Profitability map of the baseline year (2009). Output from the logging model (see Methods) implemented in Dinamica EGO software version 3.0.5 (Dinamica EGO Copyright 1998–2015 Centro de Sensoriamento Remoto / Universidade Federal de Minas Gerais–Brazil. Available at http://www.csr.ufmg.br/dinamica). Map design made in ArcMAP version 10.2 (ArcGIS Copyright 2016 Environmental Systems Research Institute, Inc. http://desktop.arcgis.com/en/arcmap/).
(TIF)

S7 Fig Simulation of road network expansion in the model. Selected cells to be logged are connected to the logging center by existing roads combined with the new unpaved ones that are built to reach these areas. Construction follows the least cost pathway directed to areas with available profitable wood volume following the harvest rules of each mode. Output from the logging model (see Methods) implemented in Dinamica EGO software version 3.0.5 (Dinamica EGO Copyright 1998–2015 Centro de Sensoriamento Remoto / Universidade Federal de Minas Gerais–Brazil. Available at http://www.csr.ufmg.br/dinamica).
(TIF)

S8 Fig Comparison of total simulated logged area [thousand hectares] after 30-years cycle for 39 National Forests under LEGAL and ILLEGAL scenarios. Number of total National Forests according to *Cadastro Nacional de Florestas Públicas* [48]. Every three bars, beginning with the green one, refer to one National Forest and its total logged area under LEGAL scenario (green), ILLEGAL (red), and the difference between both (yellow). Graph created using Microsoft Excel 2010.
(TIF)

S9 Fig Total logged wood volume per year per Brazilian State from 2009 to 2038 under ILLEGAL scenario. Maranhão and Tocantins States are omitted due to low representativeness. Graph created using Microsoft Excel 2010.
(TIF)

S10 Fig Total logged wood volume per year per Brazilian State from 2009 to 2038 under LEGAL Scenario. Maranhão and Tocantins States are omitted due to low representativeness. Graph created using Microsoft Excel 2010.
(TIF)
