# Supplementary figures and images for: Illegal logging as a disincentive to the establishment of a sustainable forest sector in the Amazon
Source: PLoS One. 2018 Dec 5;13(12):e0207855. doi: 10.1371/journal.pone.0207855 (PMC6281205; doi:10.1371/journal.pone.0207855)

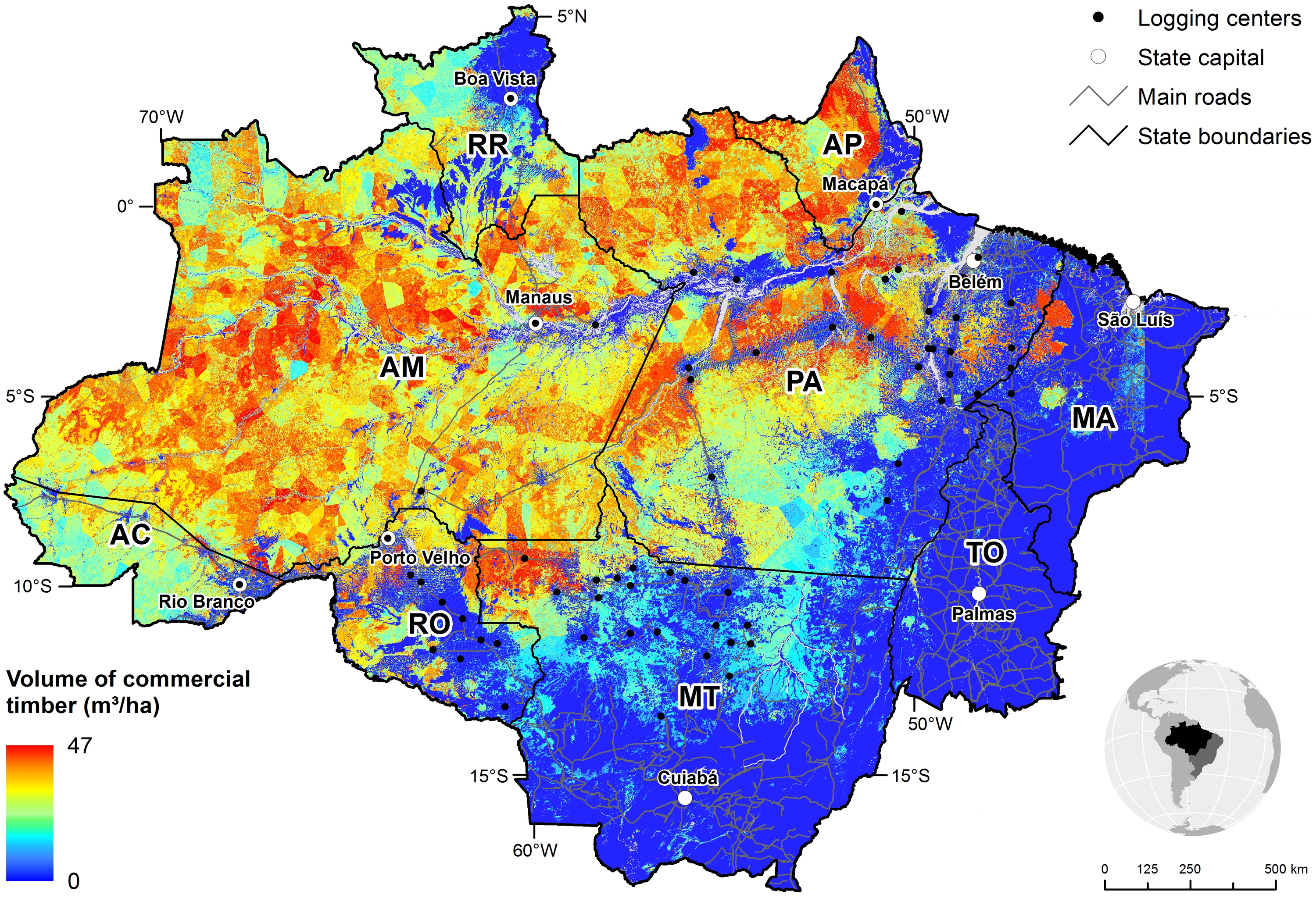

Supplement: S1 Fig — Calculated based on Merry et al. (2) (co-authors) updated with official land cover map of 2009 from PRODES/INPE (3) (Available at http://www.dpi.inpe.br/prodesdigital/prodes.php?LANGUAGE=EN&). Map design made in ArcMAP version 10.2 (ArcGIS Copyright 2016 Environmental Systems Research Institute, Inc. http://desktop.arcgis.com/en/arcmap/). (TIF) [file pone.0207855.s001.tif]

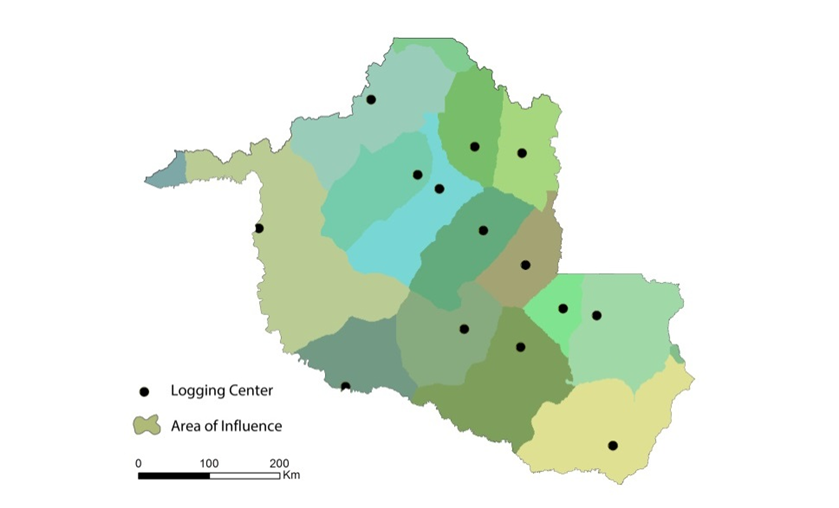

Supplement: S2 Fig — Logging centers are depicted in black spots and areas of influence with a variety of shades of green. This is an output from the logging model (see Methods) implemented in Dinamica EGO software version 3.0.5 (Dinamica EGO Copyright 1998–2015 Centro de Sensoriamento Remoto / Universidade Federal de Minas Gerais–Brazil. Available at http://www.csr.ufmg.br/dinamica). Map design made in ArcMAP version 10.2 (ArcGIS Copyright 2016 Environmental Systems Research Institute, Inc. http://desktop.arcgis.com/en/arcmap/). (TIF) [file pone.0207855.s002.tif]

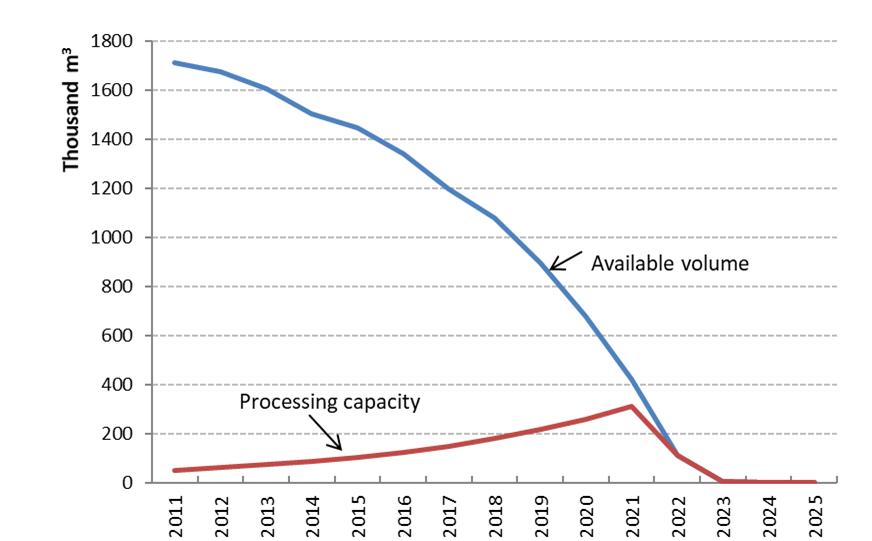

Supplement: S3 Fig — Center capacity is expressed in Thousand m3 per year (red line), following the profitable available wood volume in Thousand m3 per year (blue line). Graph created using Microsoft Excel 2010. (TIF) [file pone.0207855.s003.tif]

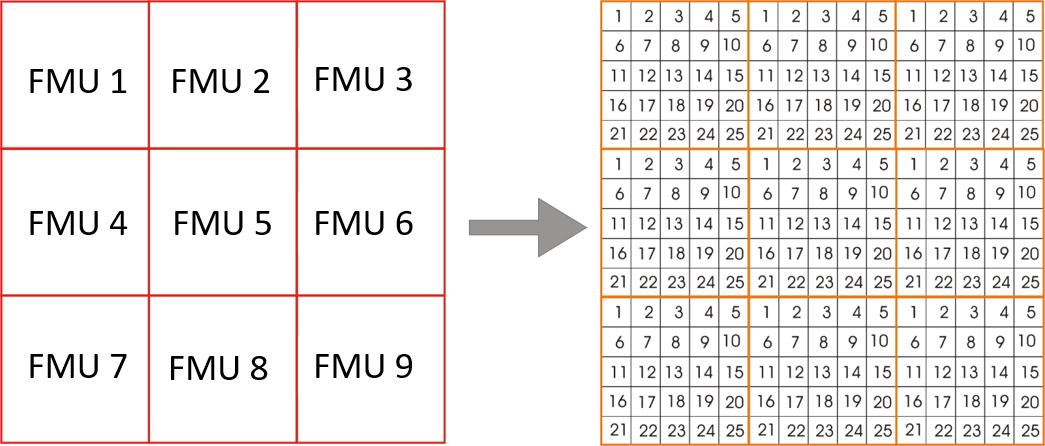

Supplement: S4 Fig — FMUs are represented as large squares with orange borders while each APU is represented by a small square. (TIF) [file pone.0207855.s004.tif]

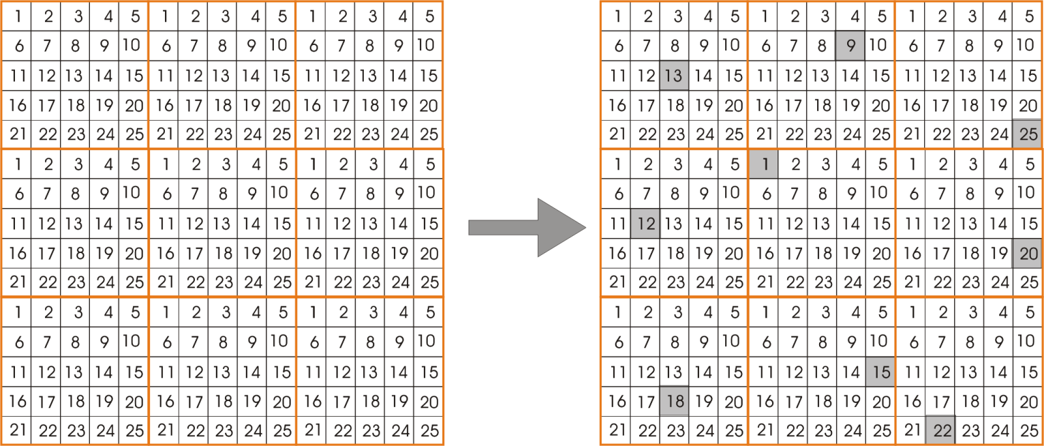

Supplement: S5 Fig — FMUs are represented as large squares with orange borders while each APU is represented by a small square. Grey small squares represent the selected APU for each FMU. (TIF) [file pone.0207855.s005.tif]

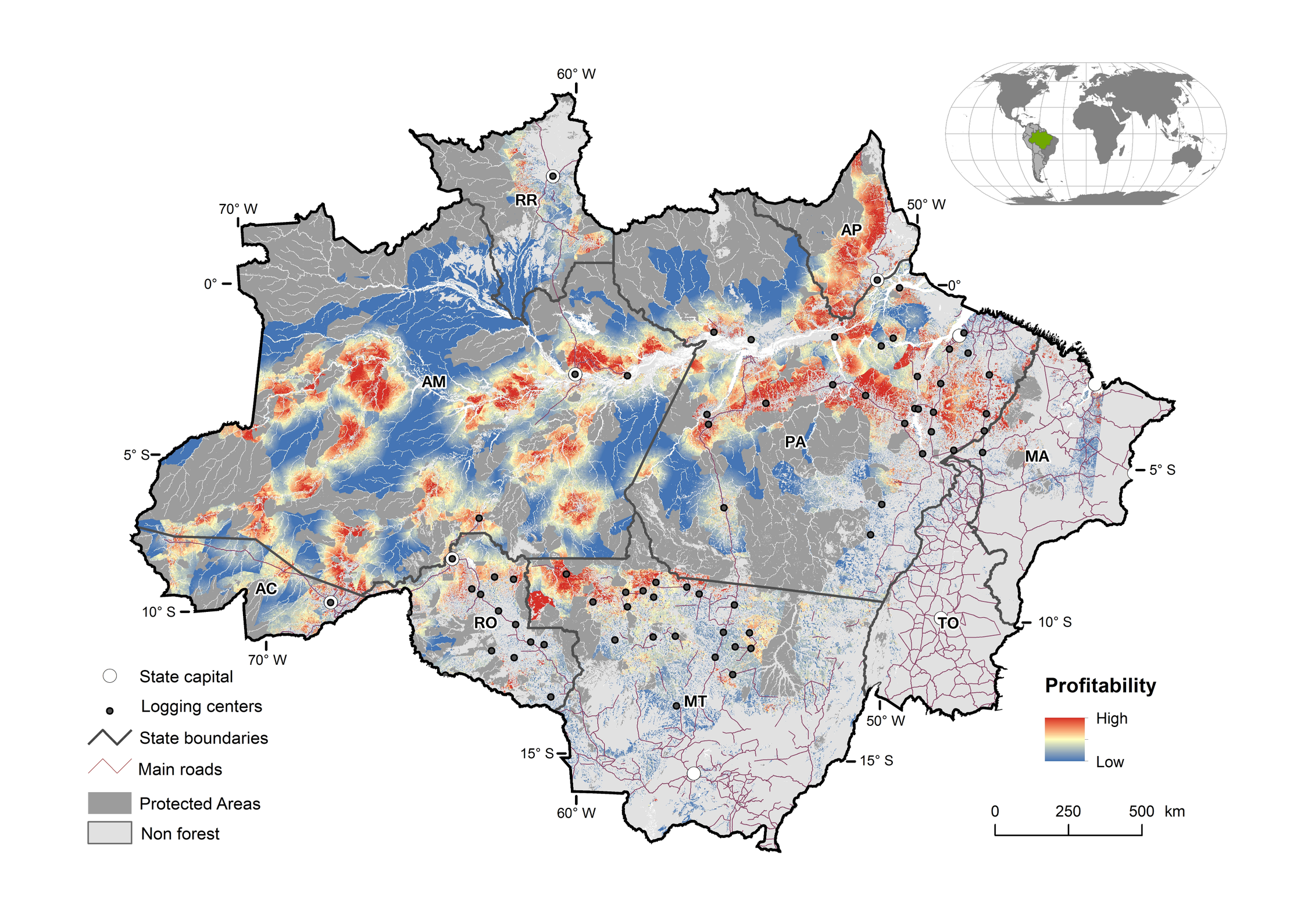

Supplement: S6 Fig — Output from the logging model (see Methods) implemented in Dinamica EGO software version 3.0.5 (Dinamica EGO Copyright 1998–2015 Centro de Sensoriamento Remoto / Universidade Federal de Minas Gerais–Brazil. Available at http://www.csr.ufmg.br/dinamica). Map design made in ArcMAP version 10.2 (ArcGIS Copyright 2016 Environmental Systems Research Institute, Inc. http://desktop.arcgis.com/en/arcmap/). (TIF) [file pone.0207855.s006.tif]

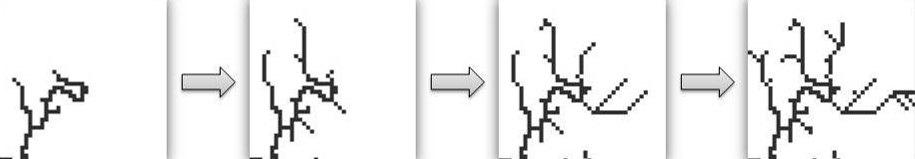

Supplement: S7 Fig — Selected cells to be logged are connected to the logging center by existing roads combined with the new unpaved ones that are built to reach these areas. Construction follows the least cost pathway directed to areas with available profitable wood volume following the harvest rules of each mode. Output from the logging model (see Methods) implemented in Dinamica EGO software version 3.0.5 (Dinamica EGO Copyright 1998–2015 Centro de Sensoriamento Remoto / Universidade Federal de Minas Gerais–Brazil. Available at http://www.csr.ufmg.br/dinamica). (TIF) [file pone.0207855.s007.tif]

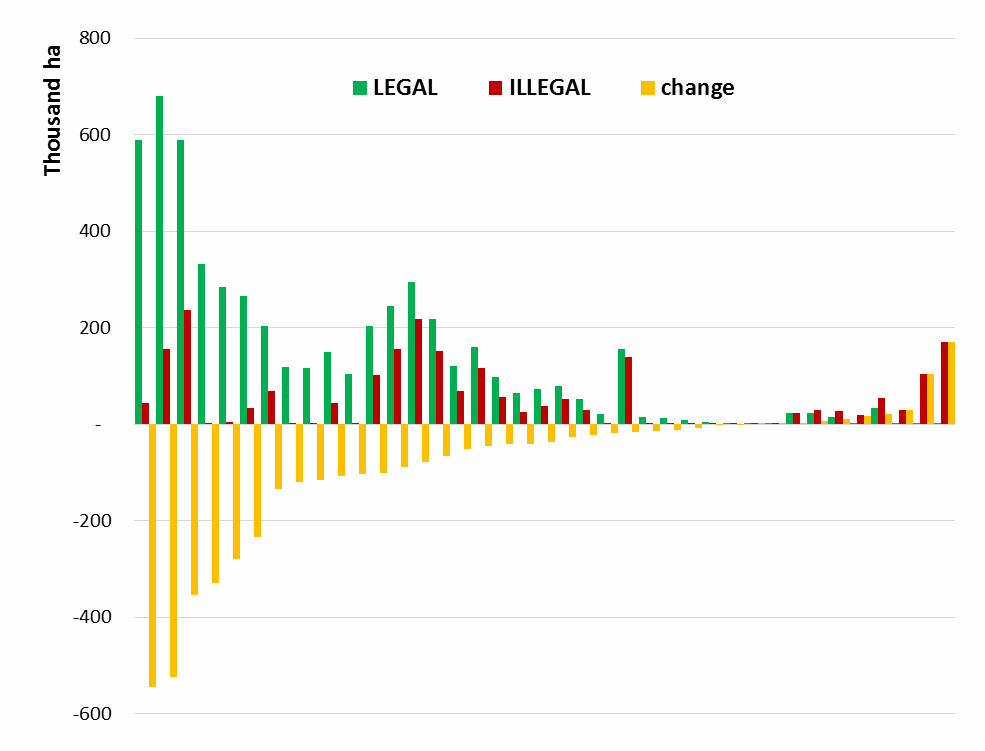

Supplement: S8 Fig — Number of total National Forests according to Cadastro Nacional de Florestas Públicas [48]. Every three bars, beginning with the green one, refer to one National Forest and its total logged area under LEGAL scenario (green), ILLEGAL (red), and the difference between both (yellow). Graph created using Microsoft Excel 2010. (TIF) [file pone.0207855.s008.tif]

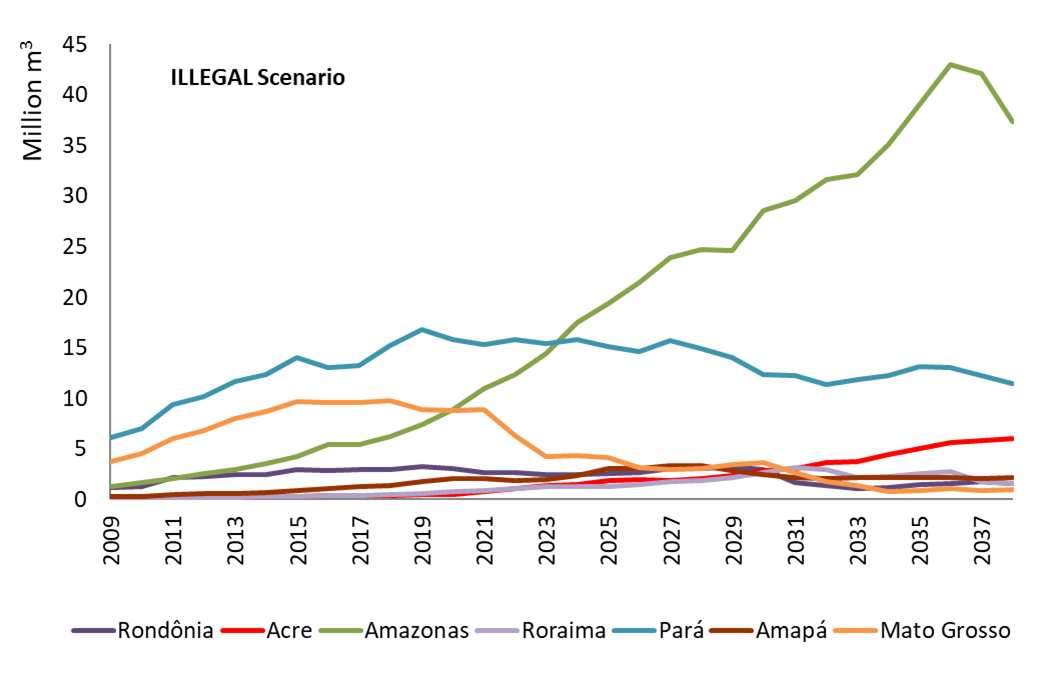

Supplement: S9 Fig — Maranhão and Tocantins States are omitted due to low representativeness. Graph created using Microsoft Excel 2010. (TIF) [file pone.0207855.s009.tif]

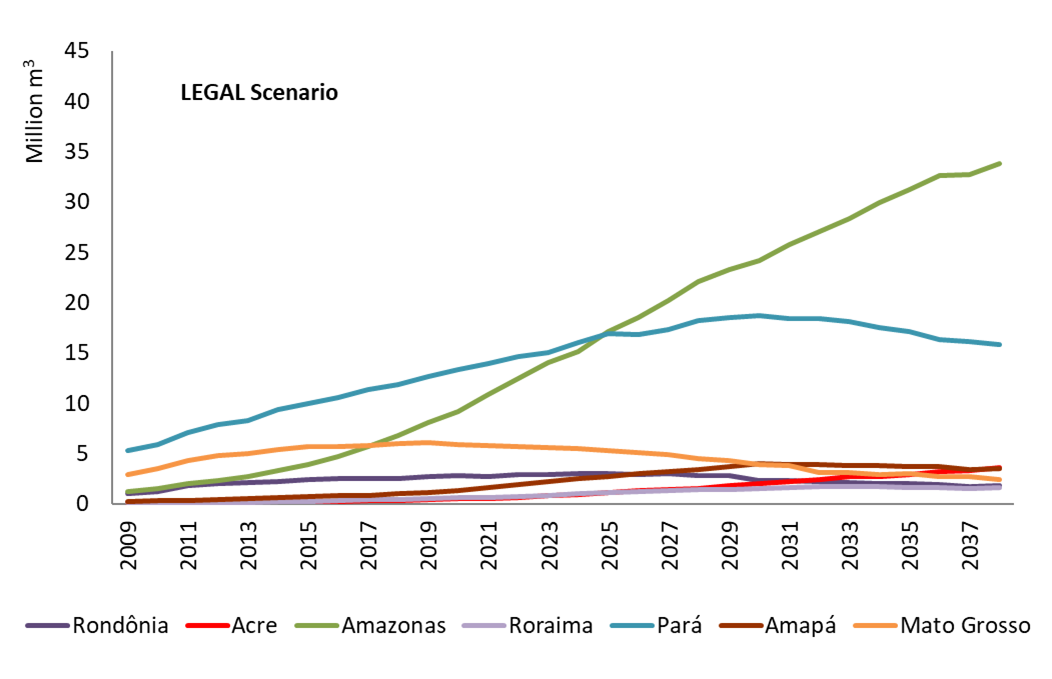

Supplement: S10 Fig — Maranhão and Tocantins States are omitted due to low representativeness. Graph created using Microsoft Excel 2010. (TIF) [file pone.0207855.s010.tif]
